# The mature anther-preferentially expressed genes are associated with pollen fertility, pollen germination and anther dehiscence in rice

**DOI:** 10.1186/s12864-015-1305-y

**Published:** 2015-02-19

**Authors:** Sheng Ling, Caisheng Chen, Yang Wang, Xiaocong Sun, Zhanhua Lu, Yidan Ouyang, Jialing Yao

**Affiliations:** College of Life Science and Technology, Huazhong Agricultural University, Wuhan, 430070 China; College of Plant Science and technology, Huazhong Agricultural University, Wuhan, 430070 China; National Key Laboratory of Crop Genetic Improvement and National Center of Plant Gene Research (Wuhan), Huazhong Agricultural University, Wuhan, 430070 China

**Keywords:** Mature anther, Microarray, Rice, RNA interference

## Abstract

**Background:**

The anthers and pollen grains are critical for male fertility and hybrid rice breeding. The development of rice mature anther and pollen consists of multiple continuous stages. However, molecular mechanisms regulating mature anther development were poorly understood.

**Results:**

In this study, we have identified 291 mature anther-preferentially expressed genes (*OsSTA*) in rice based on Affymetrix microarray data. Gene Ontology (GO) analysis indicated that *OsSTA* genes mainly participated in metabolic and cellular processes that are likely important for rice anther and pollen development. The expression patterns of *OsSTA* genes were validated using real-time PCR and mRNA *in situ* hybridizations. *Cis*-element identification showed that most of the *OsSTA* genes had the *cis*-elements responsive to phytohormone regulation. Co-expression analysis of *OsSTA* genes showed that genes annotated with pectinesterase and calcium ion binding activities were rich in the network, suggesting that *OsSTA* genes could be involved in pollen germination and anther dehiscence. Furthermore, *OsSTA* RNAi transgenic lines showed male-sterility and pollen germination defects.

**Conclusions:**

The results suggested that *OsSTA* genes function in rice male fertility, pollen germination and anther dehiscence and established molecular regulating networks that lay the foundation for further functional studies.

**Electronic supplementary material:**

The online version of this article (doi:10.1186/s12864-015-1305-y) contains supplementary material, which is available to authorized users.

## Background

Rice is a staple food for nearly half of the world’s population and a model species for monocot developmental studies [1]. Stamen is the male reproductive organ that consists of anthers and filaments. Anthers produced pollens, the male gametes, which are one of the major routes of gene flow in nature through cross-pollination [2]. In addition, anther development and pollen fertility are directly associated with rice yield in the agricultural production. The key role of male sterility in rice heterosis spurred people to investigate the regulatory mechanism of pollen development [3]. Therefore, rice anther and pollen development is an active area of research plant reproduction and crop breeding. Anther development in rice was divided into 14 stages, which was consistent with that of Arabidopsis [2,4]. To summarize, anther development initiates with stamen primordium formation, followed by the primordium differentiation to form the anther wall and pollen mother cells. Subsequently, the meiocytes undergo meiotic divisions and the anther wall degenerates. Afterward, the released microspores undergo two rounds of mitosis to develop into tri-cellular pollen and the pollen grains further accumulate starch and lipidic materials. Last, the pollen grains are released from anthers during the anther dehiscence.

Genome-wide expression analysis during rice anther development could help establish regulatory networks, and further analysis that investigates co-expressed gene groups may help identify DNA *cis*-elements and their interacting protein factors. In recent years, several transcriptome analyses of rice male reproductive organs were reported [5-8]. These transcriptomic studies using high density microarrays revealed the complexity of gene expression during anther development. The studies based on Agilent 44 K microarray or Affymetrix rice genome array showed that most rice genes were expressed in developing anthers and the expression of thousands of genes were anther-specific [5,7]. Furthermore, some reports utilized laser-microdissection to isolate microspores and the tapetum layer of developing anthers [6]. More recently, transcriptome profilings revealed genes contributing to specific aspects to meiosis and male gametophyte development [8]. Nevertheless, transcriptomic analyses of genes predominantly expressed in mature anthers instead of developing anthers were not performed.

During anther and pollen maturation, pollen metabolism, anther dehiscence, and pollen germination after pollination were key events for male fertility. Previous studies described several genes that function in anther dehiscence and pollen tube growth. The *SIZ1* gene in rice encoded a SUMO E3 ligase and was expressed in all tissues. The spikelet sterility in *siz1* mutant and *SIZ1*-RNAi lines was caused by defective anther dehiscence but not defective pollen [9]. Spikelet sterility in rice *pss1* mutants was due to a failure in anther dehiscence at the time of spikelet opening or even after its closing [10]. However, little is known about the genetic control of anther dehiscence in rice and how it is coordinated with other developmental processes in the anther and florets.

Several studies have revealed genes that played roles in the pollen tube growth such as *OsAP65*, *VGD1*, *Pi CDPK* and *MGP2* [11-14]. The *OsAP65* encoded a transmembrane protein and was expressed in various organs, and the anthers of *OsAP65* mutant developed normally until mature stage, showing defect in pollen tube elongation. *VGD1* was expressed specifically in pollen grains and pollen tube in Arabidopsis and encoded a pectin methylesterase (PME)-homologous protein that acted in cell wall modification. *vgd1* pollen tubes were unstable and burst more frequently than those of wild type. In addition, two pollen-expressed calmodulin-like domain protein kinases *Pi CDPK1* and *Pi CDPK2* in Petunia, and MALE GAMETOPHYTE DEFECTIVE 2 (*MGP2*) in Arabidopsis were involved in pollen germination and pollen tube growth. These results showed that the genes expressed in pollen were necessary for pollen grain germinate or pollen tube elongation. Although gene knockdown or knockout approaches can be used to identify gene functions, they do not illustrate the systems level molecular networks of mature anther development. The molecular mechanism concerning this specific late stage anther development was still poorly understood compared with early- or mid-term stages of the anther development.

In this study, we aimed to provide molecular insights into genes preferentially expressed in rice mature anthers by establishing their regulatory networks based on transcriptome profiling and gene co-expression analysis. Genes highly expressed in the mature stamen at higher than 4 fold levels compared with other organs in Minghui63 (MH63) or Zhenshan97 (ZS97) rice were defined as mature anther-preferentially expressed genes (*OsSTA*) [15]. Expression patterns during anther development and co-expression analyses were performed to discover the potential functions of the *OsSTA* genes. We have validated the microarrays data by quantitative real-time PCR. In addition, some genes were chosen to confirm the spatial and temporal expression by *in situ* hybridization. Finally, we have constructed *OsSTA-*RNAi transgenic plants for gene functional characterization.

## Results

### Identification and nomenclature of *OsSTA* genes

**Figure 1:**
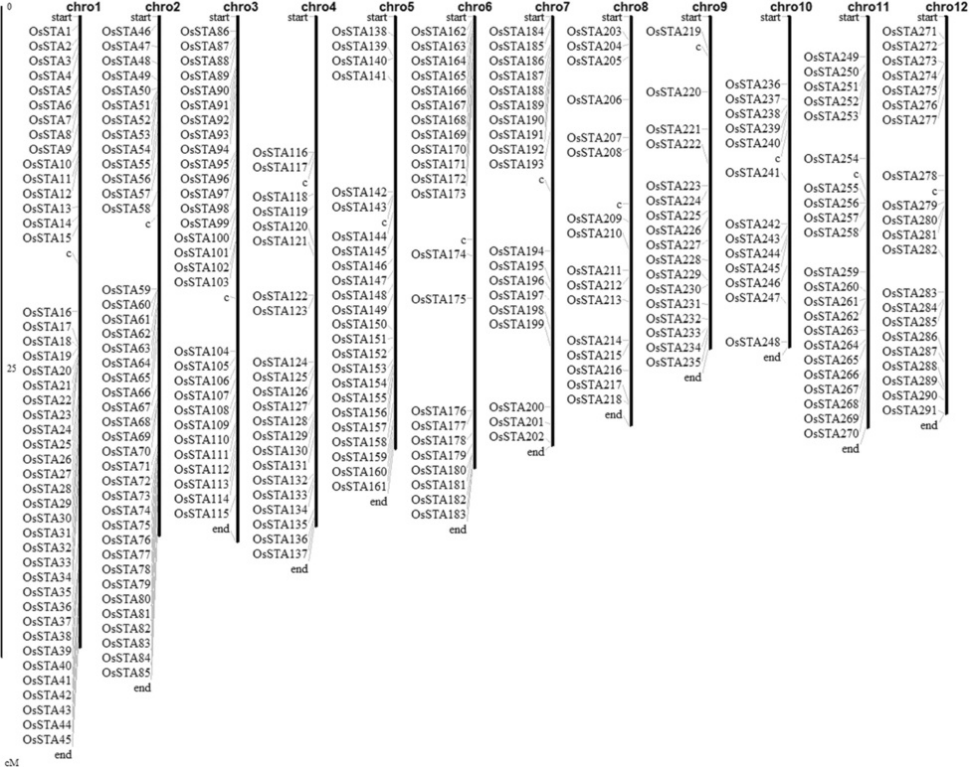
**Genomic distribution of** ***OsSTA*** **genes on rice chromosomes.** The scale on the left is in megabases (Mb). The marks with c on the chromosomes indicate the position of centromeres; the chromosome numbers are shown on the top of each bar.

**Figure 2:**
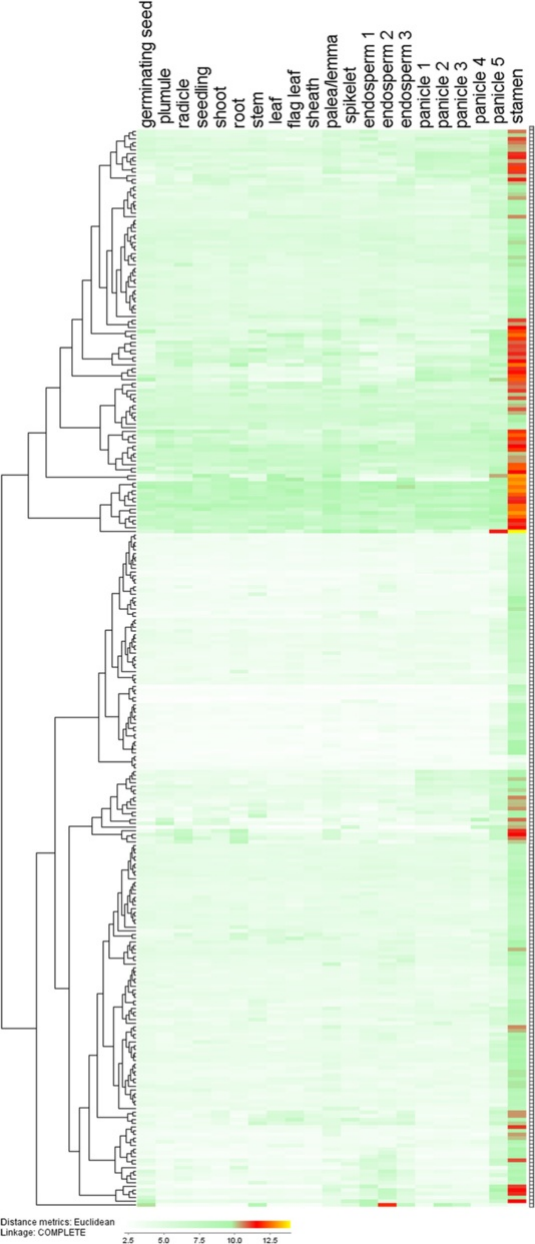
**A hierarchical clustering of 291** ***OsSTA*** **genes according to their expression patterns during rice life cycle in MH63.** Color bar at the base represents log_2_ expression values: white, representing low expression; green, medium expression; red and orange, high expression.

**Figure 3:**
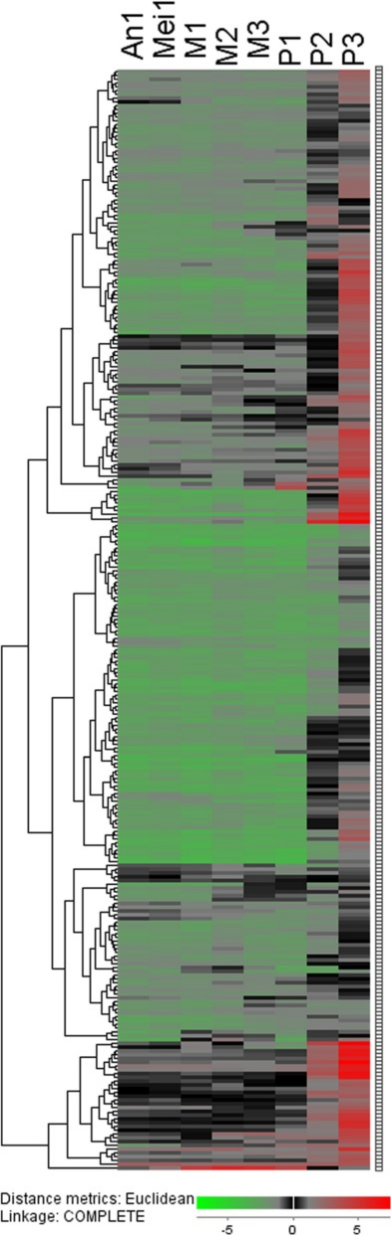
**Expression patterns of** ***OsSTA*** **genes in rice male reproductive process.** Color bar at the base represents log2 expression values: green, representing low expression; black, medium expression; red, high expression. An1: Anther in hypodermal archesporial cells forming stage; Mei1: Anther in pollen mother cells at pre-meiotic S/G2 stage; M1: Anther in pollen mother cells at meiotic leptotene stage; M2: Anther in pollen mother cells at meiotic zygotene-pachytene stage; M3: Anther in pollen mother cells at meiotic diplotene-tetrad stage; P1: Anther in uni-nucleated gametopyte stage; P2: Anther in bi-cellular gametopyte stage; P3: Anther in tri-cellular mature pollen stage.

The definition of mature anther in this experiment was that with tri-nucleus and materials accumulation of pollens, followed with the anther connective burst. The data for rice mature anther-preferentially expressed genes was cited from the results of tissue-specific expressed genes in previous study, which contain 21 tissues during the rice development [15]. Based on the data, we have identified 402 probe sets preferentially expressed in the mature anther and 4 folds higher than other tissues. During them, 83 probe sets were removed for absence of the gene IDs. The remaining 319 probe sets corresponding to 291 genes were considered as mature anther-preferentially expressed genes and were named from *OsSTA1* to *OsSTA291* according to their positions on pseudomolecules (Figure 1). The microarrays concerning the rice entire life cycle in Wang et al. 2010 [15] were used for hierarchical clustering analysis. The GSE19024 Affymetrix microarray from NCBI showed that all of the 291 genes had high transcript accumulations in mature anther. A hierarchical cluster displaying the log2 of average signal values for the 291 *OsSTA* genes was generated in MH63 (Figure 2; Additional file 1: Table S1a; Additional file 2: Table S2). The cluster suggested that all genes showed the highest expression signals in mature anther. Another microarray GSE13988 was used to confirm the expression patterns of the 291 *OsSTA* genes, which contained 8 anther developmental stages from hypodermal archesporial cells formation to tri-cellular pollen stage in Nipponbare (Fujita et al. 2010 [7]). The hierarchical clustering analysis based on this array data was carried out, using the same probe sets in MH63 (Figure 3; Additional file 1: Table S1b). The heat map showed that 85% (247/291) of the *OsSTA* genes had the highest expression levels in the anther with tri-cellular pollen. Besides, most of them had expression signals in bi-cellular pollens. Therefore, we defined the 291 *OsSTA* genes as rice mature anther-preferentially expressed genes and performed further analysis on these *OsSTA* genes. Detailed information on the *OsSTA* genes such as accession numbers, protein properties, and isoelectric points is listed in Additional file 3: Table S3, at the times we take the *OsSTA* genes sequence information in Additional file 3: files 1-3.

### Validation of the microarrays data by quantitative real-time reverse transcription-PCR

**Figure 4:**
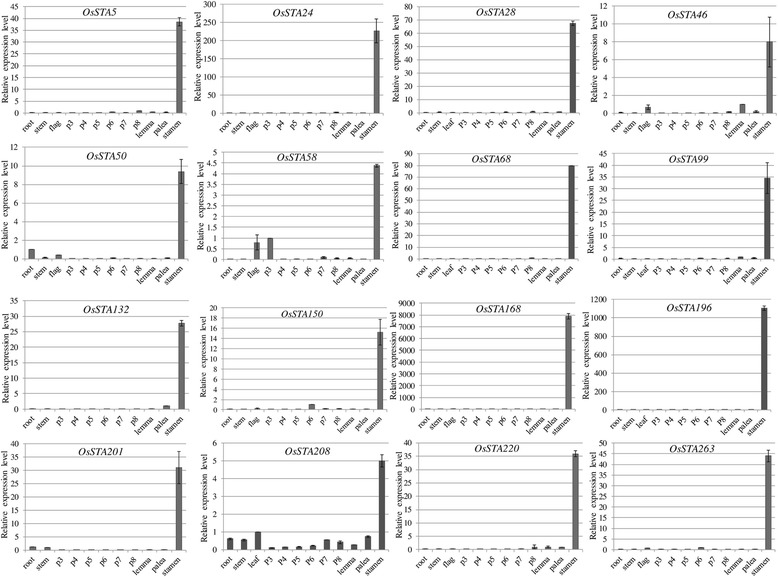
**The relative expression level of selected 16** ***OsSTA*** **genes in ZH11.** Y-axis represents relative expression values obtained using real-time PCR. X-axis depicts developmental stages as explained.

Sixteen *OsSTA* genes were randomly chosen for further validation by real-time PCR (primers details see Additional file 4: Table S4a). Various rice tissues from Zhonghua11 (ZH11) were used, includes flowering stage roots, stems and flag leaves, the panicles from archesporial cells formation to bi-cellular pollen stage (P3-P8), paleas (contains pistils), lemmas and mature anthers in tri-cellular mature pollen stage [16]. In this experiment, the ubiquitin was used for internal control gene. The results of real-time PCR showed that the expression patterns of these genes were in general agreement with the data of the microarrays (Figure 4). Remarkably, the expression level was higher in the mature anther than those in the other tissues (at least 4 fold). *OsSTA46* (*Os02g01990*), *OsSTA50* (*Os02g09530*), *OsSTA58* ( *Os02g20530*) and *OsSTA208* ( *Os08g15090*) showed 4 to 10 fold, *OsSTA5* (*Os01g08340*), *OsSTA28* (*Os01g50470*), *OsSTA68* (*Os02g43840*), *OsSTA99* (*Os03g23030*), *OsSTA132* (*Os04g52950*), *OsSTA150* (*Os05g37150*), *OsSTA201* (*Os07g47120*), *OsSTA220* (*Os09g09630*) and *OsSTA263* (*Os11g36740*) showed 10 to 100 fold, and *OsSTA24* showed more than 200 fold than other tissues. The results confirmed that these genes were mature anther-preferentially expressed genes. Furthermore, *OsSTA168* (*Os06g05710*) and *OsSTA196* (*Os07g31830*) showed more than one thousand fold than other tissues, which indicated these two genes specifically expressed in the mature anther and had no signal in other tissues.

### The spatial and temporal activities of *OsSTA* genes in rice anther

**Figure 5:**
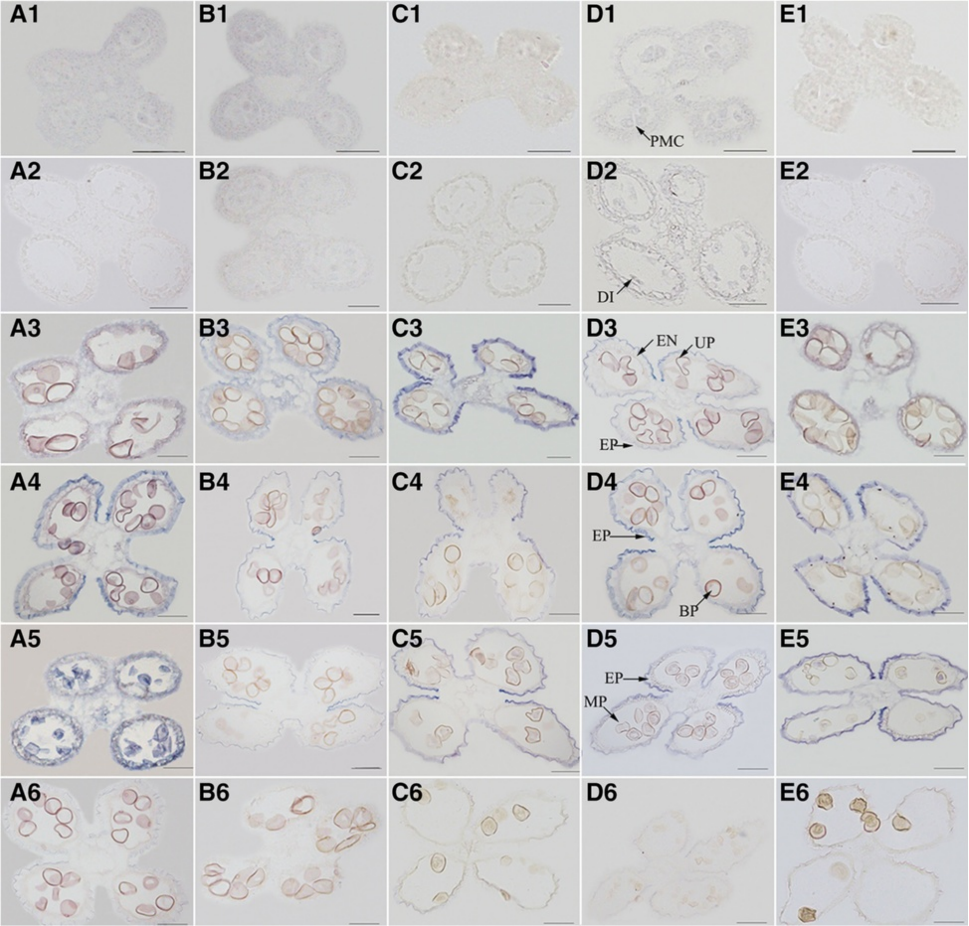
***In situ*** **localization of** ***OsSTA*** **transcript during anther development in ZH11.** A1-A6: *OsSTA28*; B1-B6: *OsSTA208*; C1-C6: *OsSTA196*; D1-D6: *OsSTA99*; E1-E6: *OsSTA68*; A1-E1: pollen mother cell stage; A2-E2: pollen mother cell meiosis stage; A3-E3: microspore stage; A4-E4: bi-cellular pollen stage; A5-E5: mature anther stage; A6-E6: sense probe control. PMC: pollen mother cell; DI: diplotene; EP: epidermis; EN: endothecium; UP: uni-nucleated pollen; BP: bicellular pollen; MP: mature pollen. Bars = 50 μm.

*In situ* hybridization was performed to examine the temporal and spatial expression patterns of *OsSTA* genes in rice anther. Five genes from real-time PCR test, including *OsSTA28*, *OsSTA68*, *OsSTA99*, *OsSTA196*, and *OsSTA208*, were selected for mRNA *in situ* hybridization. The transverse sections of roots, stems and flag leaves in flowering stage, anther from pollen mother cell stage to mature pollen stage were used for analysis (Figure 5). No signal was detected in the anthers of microspore stage using a dig oxigenin (DIG)-labeled *OsSTA* genes sense probe as a control.

The *OsSTA* genes showed similar spatial and temporal expression patterns, all of which had no detection signal in vegetative organs and early stage anthers such as pollen mother cell and meiosis stage using the DIG-labeled *OsSTA* genes anti-sense probe for research (Figure 5; Additional file 5: Figure S1). The genes expression signal in anther could be detected from microspore stage (except *OsSTA28* and *OsSTA68*) to tri-cellular mature pollen stage, and the anther wall had the transcript accumulation. More accurately, *OsSTA28* expressed in the pollens of mature anther and the anther wall from bi-cellular pollen to mature anther stage. The expression signal of *OsSTA99*, *OsSTA196* and *OsSTA208* appeared on the anther wall from microspore stage to mature anther stage, in addition, *OsSTA99* had transcript accumulation in the mature pollens. Besides, the *OsSTA68* was only expressed in anther wall from bi-cellular pollen stage to mature anther stage. Interestingly, all of the *OsSTA* genes had no expression signal in the stomium of anther. The similar expression patterns implied that the detective *OsSTA* genes might perform functions during the mature processes of the anther.

### Gene ontology (GO) analyses and functional classification

**Figure 6:**
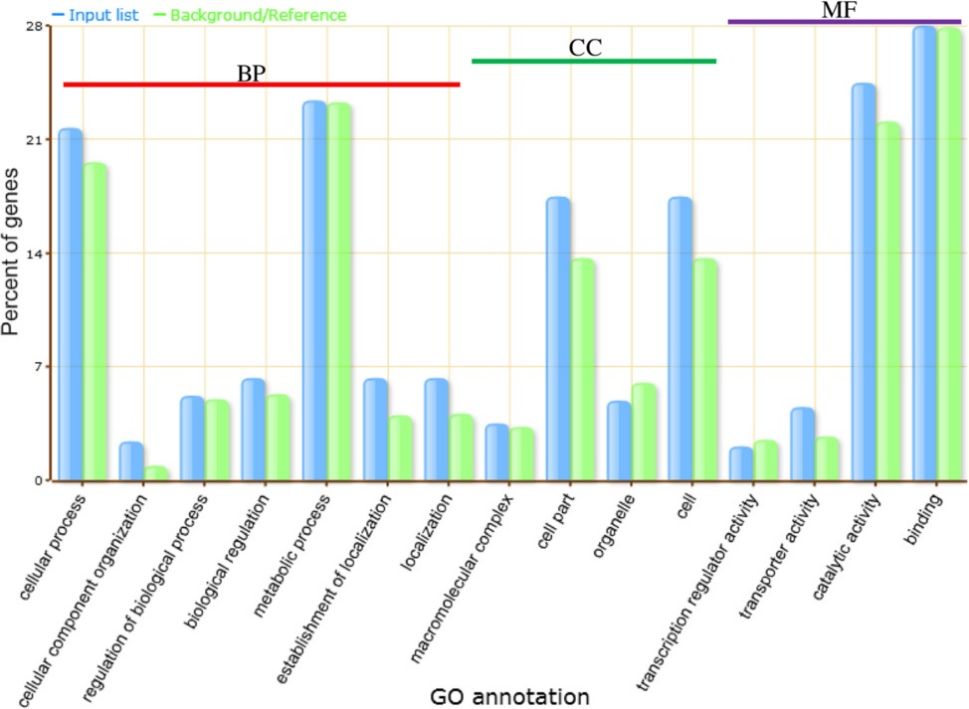
**Functional classification and enriched GO analysis of** ***OsSTA*** **genes.** X-axis represents the GO annotation and Y-axis represents the percentage of GO annotation. BP: biological process; CC: cellular component; MF: molecular function. Input list represents genes analyzed, and the references/background represents all genes in agriGO database.

AgriGO is a useful tool to analyze the GO annotations concentrating in molecular function, biological process and cellular component [17]. We thus analyzed the GO annotations of 291 *OsSTA* genes by the agriGO tools, which indicated functionality of 286 genes (Additional file 6: Table S5). These 286 genes were classified into 15 categories: cellular process, cellular component organization, regulation of biological process, biological regulation, metabolic process, establishment of localization and localization in biological process; macromolecular complex, cell part, cell and organelle in cellular component; transcription regulator activity, transporter activity, catalytic activity and binding activity in molecular function (Figure 6). During these GO annotations, the *OsSTA* genes enriched in categories of cellular process (62/286), metabolic process (67/286), catalytic activity (70/286) and binding activity (80/286). More exactly, the 67 genes belong to metabolic process were mainly involved in cellular metabolic process, macromolecule metabolic process and primary metabolic process. The 70 genes encoding proteins with catalytic activity contained 16 kinases, 28 transferases and 30 hydrolases. Further analysis showed that most of the enzymes participated in the metabolic process, in the form of compounds. These results suggested that *OsSTA* genes might play crucial roles in cellular development and metabolic processes in rice mature anthers.

### Identifying mature anther highly expressed genes and GO analyses

To search for genome-wide molecular mechanisms in rice mature anthers, we analyzed all the mature anther highly expressed genes. In Arabidopsis, rice and other organisms, co-expression analysis was used for identifying functional transcription regulators [18]. The distribution of the Pearson’s correlation coefficients (PCC) suggested that the PCC higher than 0.75 was significant for two correlated genes [19]. The 42195 PCCs of each pair for the *OsSTA* genes were calculated, during them, 32470 PCCs were higher than 0.75, which revealed that most *OsSTA* genes had similar expression pattern, therefore, we detected the co-expression genes of *OsSTA* genes to find the mature anther highly expressed genes (Additional file 7: Table S6a). Each of the *OsSTAs* was selected to identify the co-expressed genes using expression data from CREP database (http://crep.ncpgr.cn/crep-cgi/query_by_tree.cgi), and the PCCs greater than 0.8 were chosen for next step analysis by the removal of duplicated genes. Based on this, 1510 genes were found to be highly-correlated with the expression of *OsSTA* genes group (Additional file 7: Table S6b).

**Figure 7:**
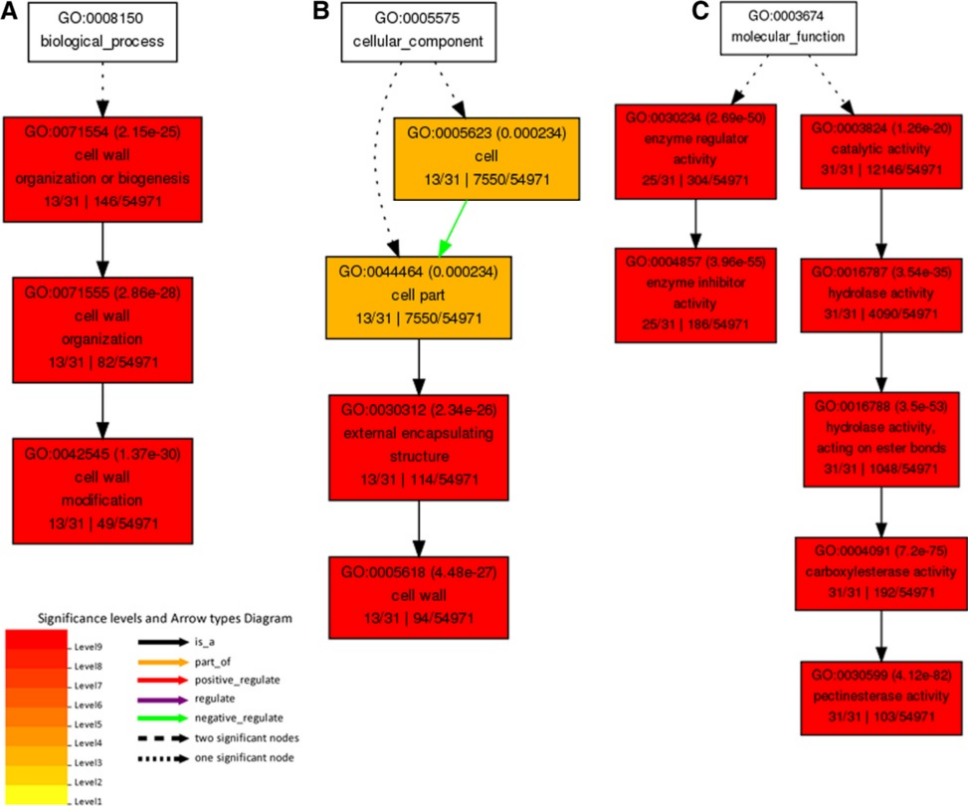
**GO annotations for genes with pectinesterase activity in the co-expression network.** The boxes in the graph list the GO identifier, the statistical significance, and the description of GO terms. The color of the box indicates the significance of the term. **A**: biological process; **B**: cellular component; **C**: molecular function.

**Figure 8:**
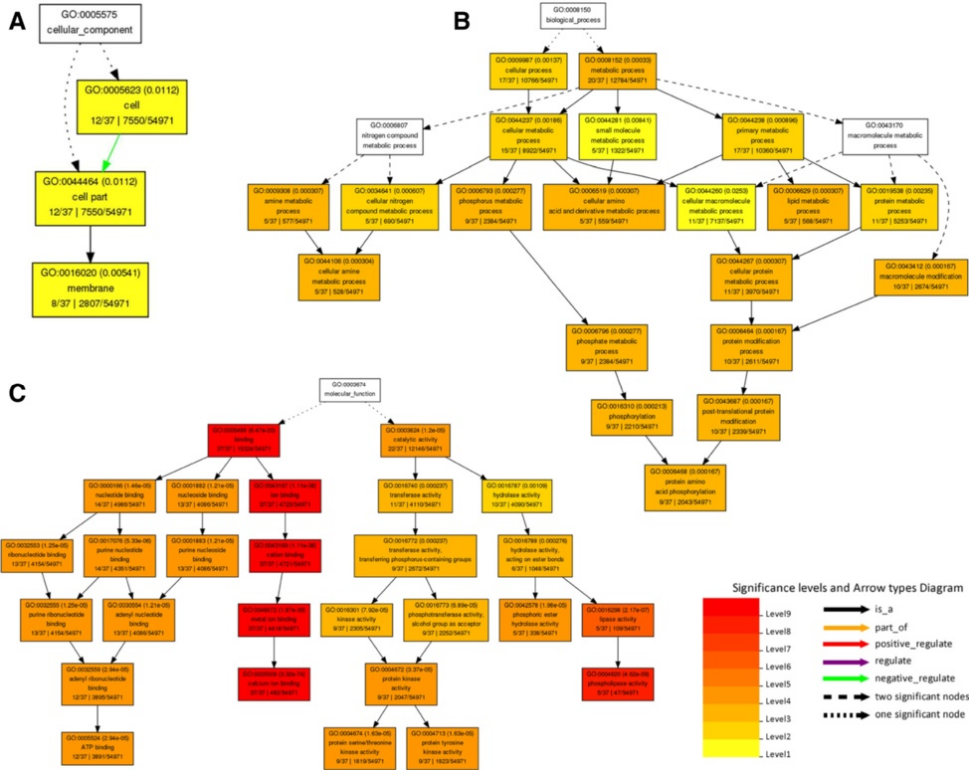
**Significant GO annotations for genes indicated in the co-expression network with a calcium ion binding activity.** The boxes in the graph list the GO identifier, the statistical significance, and the description of the GO term. The color of the box indicates the significance of the term. **A**: cellular component; **B**: biological process; **C**: molecular function.

The co-expression gene annotations were identified in the agriGO database (Additional file 7: Table S6c). Afterwards, the results involved in biological process, cellular component and molecular function were graphical (Additional file 5: Figure S2). The enriched GO annotations particularly concentrated on metabolic process, localization process, cellular process, signal transduction, biological regulation and cell wall organization or biogenesis. They encoded proteins forming macromolecular complex in cell or extracellular. The molecular functions of these genes were transporter activity, enzyme regulator activity, catalytic activity and binding. These annotations were mainly consistent with *OsSTA* genes, except for signal transduction and enzyme regulator activity, which revealed that the genes highly expressed in mature anther might function together with *OsSTA* genes. Among these genes, 31 genes were annotated with pectinesterase activity (GO: 0030599), whereas only 103 genes had this activity in the rice genome. The graphical results of gene annotations were showed in Figure 7. The graphs revealed that these genes encoded enzymes that function as modifiers of the cell wall. Also, 25 of these genes encoded proteins showing enzyme inhibitor activity and participated in enzyme regulator process. These results suggested that pectinesterase could be necessary for anther development in mature pollen. Moreover, 37 genes that code for proteins with the calcium ion binding (GO: 0005509) activities were identified, which were annotated with catalytic activity and participated in metabolic processes (Figure 8).

### Characterization of *cis*-elements in *OsSTA* gene promoters

In rice, the levels of auxin, gibberellins, ethylene and jasmonic acid in mature anthers were higher than in other tissues [20]. To investigate whether the *OsSTA* genes participated in phytohormones regulatory networks, *cis*-elements for phytohormone responsiveness at the promoter regions of *OsSTA* were identified using the PLACE database (http://www.dna.affrc.go.jp/PLACE/index.html). Based on promoter analysis, 11 *cis*-elements for abscisic acid responsiveness, 6 *cis*-elements for auxin responsiveness, 3 *cis*-elements for gibberellin response, 3 *cis*-elements for ethylene response, and 3 *cis*-elements for jasmonic acid response were used for phytohormone responsiveness research. As a result, 251 genes had the *cis*-elements that respond to phytohormones, among which 209 genes had more than one phytohormone response *cis*-elements. It was interesting that 107 genes had abscisic acid response elements in their promoters, 138 gene promoters had auxin response *cis*-elements, 141 genes were predicted to respond to gibberellin, and 192 gene promoters had jasmonic acid responsive elements. However, no gene has *cis*-elements for ethylene and cytokinin (Additional file 8: Table S7). Therefore, the results revealed that most of the *OsSTA* genes may be regulated by the phytohormone abscisic acid, auxin, gibberellins and jasmonic acid in mature anthers. Additionally, individual *OsSTA* gene promoter analysis showed that *OsSTA28* and *OsSTA208* had the abscisic acid, auxin, and jasmonic acid responsive elements; *OsSTA99* and *OsSTA196* might respond to gibberellins and jasmonic acid; *OsSTA68* possessed auxin, gibberellins and jasmonic acid responsive elements.

### Reduced expression of *OsSTA* results in male sterility and defect of pollen germination in *OsSTA* RNAi transgenic plants

To study the functions of *OsSTA* genes in rice anthers, we employed methods of dsRNA-induced RNA interference according to published procedures [21-23]. Three genes (*OsSTA28*, *OsSTA99* and *OsSTA208*) were selected for RNAi, and the gene-specific coding sequence was chose for gene-specific interference.

**Figure 9:**
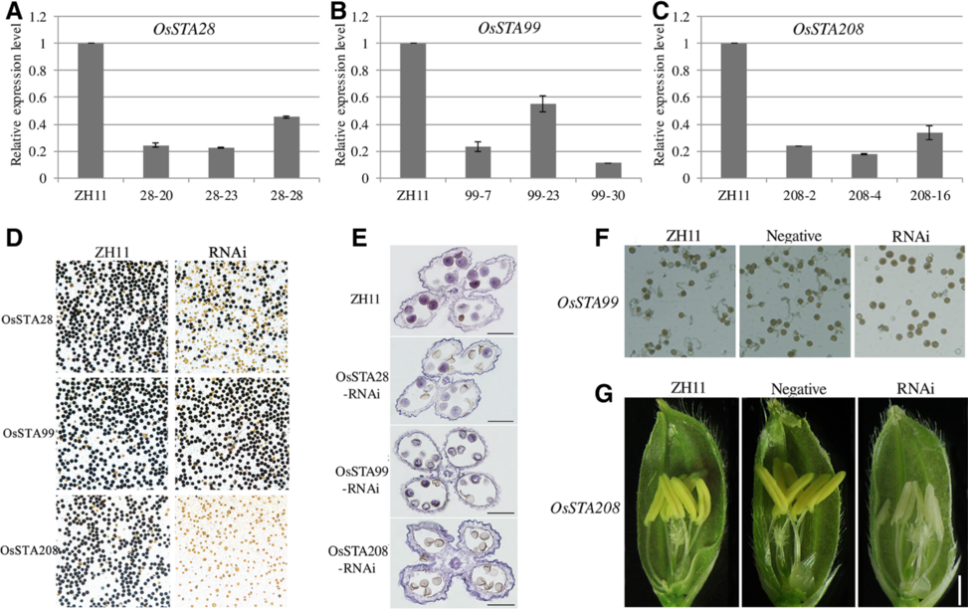
**Expression analyses and phenotypic description of** ***OsSTA*** **RNAi plants. A-C**: Real-time PCR analysis confirmed the suppressed expression of *OsSTA* genes in independent *OsSTA* transgenic lines. Anthers at mature pollen stage were analyzed. **D**: KI-I_2_ staining of wild-type pollen and mutant pollen. **E**: cross sections of wild-type and mutants anthers in mature pollen stage. **F**: *In vitro* germination of wild-type and *OsSTA99* RNAi pollen. The mutant pollen does not germinate normally. **G**.: The pollen of *OsSTA208* RNAi plants appears to be white compared with that of wild type at flowering stage. Negative represents plants without transgene. Bars = 50 μm in **(D)** to **(F)** and 2 mm in **(G)**.

After transforming dsRNA into rice protoplasts, more than 30 regenerated transgenic plants per *OsSTA* gene were obtained. Among the T_0_*OsSTA*-RNAi transgenic plants, the positive transgenic plants showed 11.3% to 85.6% decreased expression level of *OsSTA* compared with the WT (Additional file 5: Figure S3). Three positive transgenic T_0_ lines of *OsSTA* genes (*OsSTA28-20*, *28–23* and *28–28*; *OsSTA99-7*, *99–23* and *99*–*30*; *OsSTA208-2*, *208*–*4* and *208–16*) with reduced expression of *OsSTA* (2–8 fold) were selected for further analysis. The expression levels of *OsSTA* genes in these RNAi T_2_ plants were stably repressed (Figure 9 and Additional file 5: Figure S4). The defects of pollen and anther in *OsSTA* RNAi T_2_ plants were observed (Figure 9). Statistical analyses of pollen fertility and germination data in *OsSTA* RNAi and wild-type (WT) ZH11 plants were shown in Table 1.

**Table 1:** **The pollen fertility and pollen germination rate statistics of** ***OsSTA*** **genes RNAi T**_**2**_ **plants**

**Gene**		***OsSTA28***			***OsSTA99***			***OsSTA208***		
**Line**	**ZH11**	**28-20**	**28-23**	**28-28**	**99-7**	**99-23**	**99-30**	**208-2**	**208-4**	**208-16**
Fertility%	84.5 ± 6.5	31 ± 13.7**	32 ± 12.5**	38 ± 16.2**	78.5 ± 7.4	80.5 ± 10.8	74 ± 9.7*	7 ± 3.2**	8 ± 7.5**	5 ± 4.4**
Germination%	81 ± 5.8	/	/	/	33 ± 2.5**	32 ± 4.8**	38.5 ± 6.3**	/	/	/

“/” indicated the experiment not done in the lines. * t test, with P < 0.05; ** t test, with P < 0.01.

In *OsSTA28* RNAi rice plants, the anther and pollen developed normally from hypodermal archesporial cells to early bi-cellular pollen, but iodine staining of mature pollen showed that more than half were sterile (Figure 9D). In addition, cross sections of mature anthers of *OsSTA28* RNAi plants revealed that sterile pollend were shrunken and undyed compared to fertile pollens (Figure 9E). *In situ* hybridization showed that *OsSTA28* was expressed in tri-cellular pollen and anther walls after microspores underwent first mitosis. Protein conserved domain analysis showed that OsSTA28 contained RRC1, FYVE, and BRX domains. The previous study revealed that *FAB1A* and *FAB1B* encoded proteins with the FYVE domain and played key roles metabolic processes from bi-cellular to tri-cellular pollen transition in Arabidopsis [24]. According to these results, we speculated that *OsSTA28* might be a regulator of pollen fertility by influencing the unknown metabolic materials in rice.

Compared with WT anthers, anthers from *OsSTA208* RNAi plants were relatively white, their pollens were only 5-8% fertile (Table 1). Most of the pollens were no dye based on iodine stained mature pollen, and also these pollen grains failed to germinate (Figure 9D). Histological section also showed that the pollens of *OsSTA208* RNAi plants were empty and shrunken inside the mature anther (Figure 9E). It was surprising that *OsSTA208* was only expressed in the anther wall instead of pollen from microspore stage to mature anther stage by *in situ* hybridization detection. Therefore, we speculated that down-regulating expression of *OsSTA208* in anther wall might interrupt cross-talks between pollen and anther wall, which could be critical for pollen fertility. Conserved domain analysis showed that OsSTA208 possessed DYW_deaminase, four repeats of PPR, and two DAGK (diacylglycerol kinase) domains*.* Using the RNAi method, the rice DAGK family was previously found to function in regulating abiotic and biotic stresses through different signaling pathways [25]. Thus, it was possible that *OsSTA208* participated in stress response in the anther wall for regulating pollen fertility.

**Figure 10:**
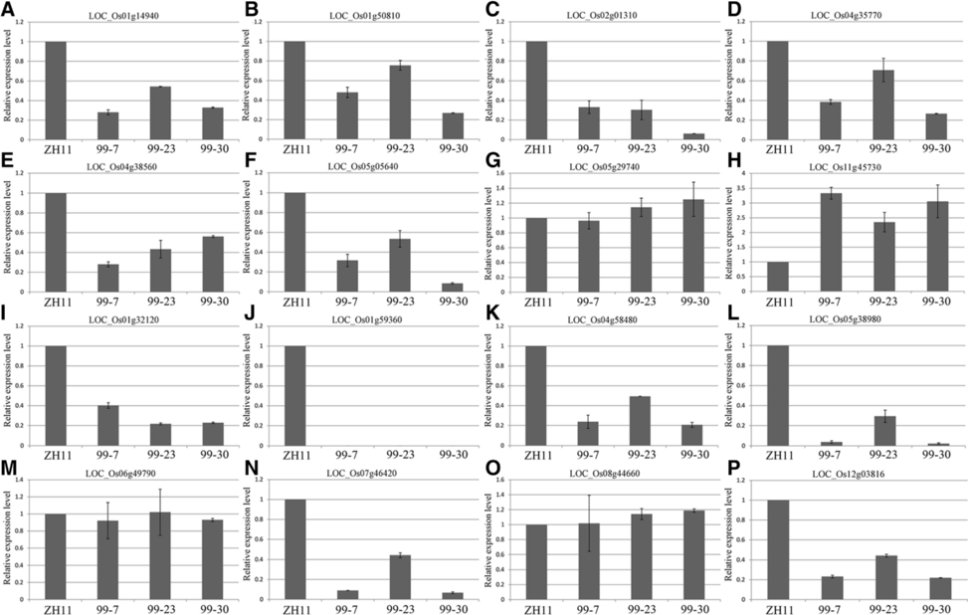
**Real-time PCR analysis confirmed altered expressions of the pectinesterase and calcium ion binding genes in the** ***OsSTA99*** **RNAi lines.** Y-axis represents relative expression values obtained using real-time PCR. X-axis represents the anthers samples at the mature pollen stage. **A-G**: pectin methylesterase activity genes; **H**: pectin methylesterase inhibitor activity genes; **I-P**: calcium ion binding activity genes.

Different from *OsSTA28* and *OsSTA208,* the anther and pollen of *OsSTA99* RNAi plants appeared to show a WT phenotype, and the pollen fertility of RNAi plants was almost identical to WT based on statistical analysis of iodine stained mature pollen (Table 1). However, most pollen grains of transgenic plants failed to germinate *in vitro* under the same culture condition (Figure 9F). *OsSTA99* was preferentially expressed in mature anthers and the *in situ* hybridization showed that *OsSTA99* was expressed in tri-cellular pollen and anther walls. Protein conserved domain analysis showed that OsSTA99 possessed only an Apt1 domain*.* Several prior reports concerning Apt1 and its homologs (SABRE and KIP) revealed that these proteins were involved in membrane trafficking and were required for the high secretory demands of tip growth in pollen tubes or roots [26,27]. It is thus reasonable to suggest that *OsSTA99* may function in regulating pollen germination and pollen tube growth by mediating material transportation or signal transferring. In previous studies, Ca^2+^ and pectinesterase were shown to be involved in pollen grain germination and pollen tube elongation. Thus 8 pectinesterase genes and 8 calcium ion binding genes were selected from the co-expression network to detect their expression levels in *OsSTA99* RNAi plants (primers details see Additional file 4: Table S4b). The expression levels of most pectinesterase genes were changed in RNAi plants compared with the WT. Among them, *Os11g45730* showed increased expression, whereas the other 6 genes showed decreased expression (Figure 10). While the 6 tested genes with calcium ion binding activity showed decreased expression, the other 2 genes did not change their expression levels. It was inferred that *OsSTA99* controlled pollen germination and pollen tube growth by regulating the expression of Ca^2+^ and pectinesterase relative genes in rice.

Taken together, based on the *OsSTA* gene expression and observed phenotypes of *OsSTA* RNAi lines, we concluded that *OsSTA* genes likely functions to ensure pollen grain germination or pollen tube growth.

## Discussion

### *OsSTA* genes could be essential for rice male fertility by mediating pollen metabolism

At mature anther stage, the pollen grain maturation was accompanied with the accumulation of starch, lipid and secondary metabolites. These metabolic activities are important for male gametophyte fertility. Here, the GO annotations analysis of 291 *OsSTA* genes revealed that the largest group consisting of 70 genes had “enzymatic activity”, and 67 genes took part in “metabolic process”. It was notable that 16 *OsSTA* genes belonged to the “kinase-like genes”. In the past decades, plant kinases were found to be vital for pollen fertility. *VPS34* encoded a phosphatidylinositol 3-kinase in Arabidopsis, and *vps34* mutants showed male gametophyte abnormality with large vacuoles and no nuclear in pollen grains in the mature pollen stage, indicating *VPS34* was essential for pollen maturation [28]. Moreover, the metabolic activities of mature pollen influenced pollen grain germination. For example, Alpha-Glucan water dikinase (GWD) was a key enzyme in tomato that controlled the phosphate content of starch and the starch degradation. In *gwd* mutants, the pollens showed excess starch accumulation and soluble sugars reducing phenotypes, leading to a reduction in pollen germination, which indicated the key role of starch metabolism for pollen germination [29].

In this study, the gene knockdown experiment was performed to determine the function of *OsSTA* genes in male fertility. We have found that pollen viability was reduced in *OsSTA28* and *OsSTA208* RNAi plants and their pollens showed varying degrees of debility. It was reported that *OsNek3* (*OsSTA192)* was preferentially expressed in mature anthers and functioned in male fertility of rice [30]. The knockout mutant of *OsNek3* did not show obvious pollen-defective phenotype. However, the over-expression of *OsNek3* caused a peculiar pollen structure; the outer cell wall of pollen grains fused together. Meanwhile, *OsPDC3* (*OsSTA202*) was a mature pollen-specific gene and encoded a pyruvate decarboxylase that may be required in pollen energy metabolism. Over-expression of *OsPDC3* caused increased enzyme activity in rice [31]. Here, the conserved domains and GO annotations analysis showed that OsSTA192 (OsNek3) and OsSTA202 (OsPDC3) had the kinase activity, and *OsSTA28*, *OsPDC3* and *OsNek3* were included in metabolic process genes group. Therefore, among the *OsSTA* genes, the kinases and metabolism related genes might play key roles in pollen fertility. It will be essential to further dissect their functions and learn their regulatory relations in the network of anther maturation in rice.

### *OsSTA* genes might regulate pollen germination and pollen tube growth by interaction with Ca^2+^ or pectinesterase

In this study, the identification of *OsSTA* co-expressed genes showed that 31 genes with “pectinesterase activity” and 37 genes with “calcium ion binding activity” were highly expressed in the mature anther. These genes annotated with the pectinesterase activity were regarded as functional factors during pollen tube growth in previous reports [32-35]. The pectin esterification played an important role in determining the mechanical properties of the pollen tube cell wall during tube elongation [32]. The degree of pectin esterification in different pollen tube location was controlled by competitive effect of pectin methylesterase (PME) and pectin methylesterase inhibitor (PMEI) [33,35]. It was reported that the interactions between PMEs and PMEIs regulated the properties and distributions of pectin [34]. The activity of PMEs was inhibited in the presence of PMEIs at the apical region of pollen tube, while the activity of PMEs in the sub-apical region of pollen tube depended on both exocytosis and endocytosis [34]. Pectinesterase genes were highly expressed in mature anther, whereas they changed expression patterns in *OsSTA99* RNAi plants. Detailed analysis found that 6 down-regulated genes were defined as *PME* based on other reports, while the up-regulated gene was *PMEI* [34]. On the basis of the results, we hypothesized that the *OsSTA99* might be located in the pectinesterase regulatory network for pollen tube growth, and coordinate the expression of *PMEs* and *PMEIs* in different pollen tube growth stage.

In our study, the genes encoding calcium ion binding activity proteins were highly expressed in mature anther, and recently, the mechanisms and functions of calcium in pollen tube growth have been extensively reviewed [36]. The tip-focused calcium gradient, calcium oscillation, Ca^2+^ sensing and flowing are essential for pollen grains germination and tube growth [36]. In Arabidopsis, a calcium-dependent protein kinase CPK32 interacts with calcium channel CNGC18 and activates it to regulate the pollen tube growth [37]. The proteins phosphatase 1 and 2A are involved in the regulation of Ca^2+^ uptake across the plasma membrane in exocytotic activities and in the biosynthesis of cell wall components that control pollen tube development in *Picea wilsonii* [22]. The *CBL1* and *CBL9* encode calcineurin B-like (CBL) proteins, which act as Ca^2+^sensor to regulate pollen germination and tube growth in Arabidopsis [38]. In *OsSTA99* RNAi plants, most of the “calcium ion binding activity” genes showed down-regulated expression, prompting us to suppose that *OsSTA99* may be related to Ca^2+^ regulating pathway in pollen tube growth. It is useful to further clarify how *OsSTA99* regulates *PMEs* and calcium ion related gene expression and elucidates the mechanism of pollen tube elongation.

The gene expression patterns in co-expression network implied that the *OsSTA* genes might be involved in Ca^2+^ or pectinesterase regulating pathway during the pollen germination or pollen tube growth. It would provide the clues to find new regulatory genes and explain the molecular mechanisms of pollen germination and pollen tube growth.

### *OsSTA* genes might function in anther dehiscence in response to phytohormones

It is well known that the anther dehiscence is the final and necessary step for anther development, resulting in the release of pollen grains to pollination, fertilization, and seed set. Recently, studies on a certain number of dehiscent mutants have shown that phytohormones contributed to the control of anther dehiscence. In the *tir1 afb1 afb2 afb3* quadruple auxin receptor mutants of Arabidopsis, anther dehiscent was defective as the endothecial lignification occurred prematurely before tapetum degeneration [39]. The *DAD1* gene in Arabidopsis encodes a lipase-like protein that catalyzes the production of free LA from cellular lipids as the first step in jasmonic acid biosynthesis. The stomium of anther in *dad1* mutant fails to open and the elongation of the filament is delayed, while the defects are rescued by the exogenous application of jasmonic acid or linolenic acid [40]. *HvGAMYB* is a transcription factor that first identified in barley aleurone cells. It is upregulated by gibberellin, and the anthers of transgenic plants overexpressing *HvGAMYB* are male sterile due to a failure in dehiscence with the stomium remaining intact [41]. The previous reports indicated that genes responding to auxin, gibberellins and jasmonic acid were involved in anther dehiscence.

Here, the identification of *cis*-elements in *OsSTA* promoters showed that a majority of *OsSTA* genes could respond to phytohormones. Coincidently, the detailed analysis indicated that the promoters of the largest group consisting of 192 genes had jasmonic acid responsive elements, meanwhile, 138 genes had the auxin response factor, 141 genes could respond to gibberellin. Thus, it appears that the *OsSTA* genes could regulate anther dehiscence by involving phytohormone regulatory network during anther dehiscence.

During anther dehiscence process, anther walls are indispensable for successful pollen grain release. Recently, a mathematical model describing the biomechanics of anther opening incorporates the bilayer structure of the mature anther wall, which comprises the epidermis and the endothecium. The model describes and demonstrates how epidermal dehydration in association with the thickened endothecium to drive anther opening and pollen release [42]. Also, there have been some reports of dehiscence mutants resulting from defects of secondary wall thickening and epidermis change [43-45]. In the Arabidopsis *myb26* mutant, the endothecium fails to expand and secondary thickening, which is seen in the WT anther endothecium, fails to occur. Overexpression of MYB26 results in ectopic secondary thickening in the epidermis [43,45]. In our study, the *in situ* hybridization showed that 5 tested *OsSTA* genes were expressed in the anther wall after the meiosis or first mitosis, which is the time of secondary thickening in the endothecium [43]. Meanwhile, these *OsSTA* genes had no expression signal in the stomium. This tissue was derived from the differentiation of epidermal cells, which did not undergo secondary thickening. Moreover, promoter analysis of these 5 *OsSTA* genes showed that they had auxin, gibberellins and jasmonic acid responsive elements. Therefore, we hypothesized that some *OsSTA* genes might be involved in anther wall development and anther dehiscence through their roles in phytohormone regulation.

## Conclusion

In conclusion, microarray expression profiling was used to investigate the mature anther preferentially expressed genes in rice. Using GO enrichment analysis and *cis*-elements identification in *OsSTA* gene promoters we showed that the *OsSTA* genes may involve in the anther maturation through different processes, such as pollen germination regulation and pollen tube growth by interaction with Ca^2+^ or pectinesterase, or participated in anther dehiscence through response for phytohormones. The gene knockdown experiment showed the male sterility and defect of pollen germination in *OsSTA* RNAi transgenic plants when the gene expression level reduced. All of the researches will provide the basis for understanding the mechanism of *OsSTA* genes in rice anther growth. The findings in our work would be useful in selecting candidate genes for functional research of *OsSTA* members during the anther maturation in rice.

## Methods

### *OsSTA* genes identification and chromosomal localization

The tissue-specific expressed gene data was used to identify the mature anther-preferentially expressed genes in the entire life cycle of rice, and the specifically expressed genes in mature anther were selected for analysis [15]. By eliminating the unannotated genes in Rice Genome Annotation Project database, the remaining ones were considered as mature anther-preferentially expressed genes. Detail information of the *OsSTA* genes was procured from MSU, KOME and Pfam database, including accession number, Pfam domain, chromosomal location, ORF length, introns number and protein parameters.

Each of the *OsSTA* genes was mapped on rice chromosomes according to their positions available in Rice Genome Annotation Project database. The distribution of *OsSTA* genes was drawn by MapInspect (http://www.plantbreeding.wur.nl/UK/software_mapinspect.html).

### Expression analysis of *OsSTA* genes in rice

Expression profiles of *OsSTA* genes in rice 21 tissues and anthers at 8 anther developmental stages for MH63 and Nipponbare were extracted from GSE19024 and GSE13988 Affymetrix rice microarray in NCBI database [7,15]. The gene average expression level of biological replicates for each sample was used for analysis. Expression values of each gene were logarithmed in Microsoft excel, and cluster analyses were performed using J-express 2011 with euclidean distances and hierarchical cluster method of “complete linkage”. When more than one probe set was available for one gene, the higher signal value of the probe sets was used for analysis.

### Identification of correlated genes and GO analyses

First, we computed the PCCs for all pairwise relationships between the 112 *OsSTA* genes in two sets of transcriptomes comprising a total of 190 microarray experiments by R-2.14.1 project. Then, the co-expression data were downloaded from the CREP database with PCCs greater than 0.8. By getting all the correlated genes together and eliminating the repeat genes, 1282 genes were used for the GO analysis.

GO enrichment was performed by Singular Enrichment Analysis (SEA) tool in agriGO database (http://bioinfo.cau.edu.cn/agriGO/analysis.php) with default parameters using the rice MSU6.1 genome annotation as background. Statistical significance was determined using Fisher’s exact test and Yekutieli multi-test adjustment.

### Quantitative real-time PCR

The cDNAs were amplified with gene-specific primers using Primer5 software. An ubiquitin gene, which showed constant expression in every sample, was chosen as an internal control for data normalization. Total RNA was isolated using RNAiso (Takara) and treated with RNase-free DNaseI (Takara) for 30 min to eliminate possible contaminating DNA. First strand cDNA was synthesized from total RNA with an oligo(dT)18 primer in a 20 μl reaction (diluted to 40 μl before use) using an M-MLV Reverse Transcriptase (Promega) according to the manufacturer’s instructions.

Real-time PCR was performed in a 10 μl volume containing 5 μl 2 × SYBR®Premix Ex Taq ™ (TaKaRa), 0.5 μl of Rox Reference Dye II (Takara), 1 μl of the cDNA sample, 2 μM of each gene-specific primer. The PCR conditions were as follows: 95 °C for 3 min, 40 cycles of 95 °C for 5 s, 60 °C for 34 s. Three replicates were used for each sample. Reaction was conducted on ABI StepOne^™^ Real-time PCR instrument (Applied Biosystems). We analyzed the relative transcript abundance using 2-ΔΔCT method, and the tissue with the highest expression signal except stamen was regarded as 1.

### *In situ* hybridization

Materials for *in situ* hybridization were sampled and immediately fixed in RNase-free FAA solution (4% formaldehyde, 10% acetic acid, 50% ethanol). The materials of roots, stems and flag leaves in flowering stage, anthers from pollen mother cell stage to tri-cellular pollen stage were used for analysis [16]. For the probe synthesis, the fragments used for *in situ* hybridization were amplified using the same primers with real-time PCR and sub-cloned into pGEM-T vector. Dig oxigenin-labeled RNA probes were prepared using a DIG Northern Starter Kit (Roche). T7 and SP6 RNA polymerase were used to generate the sense and antisense RNA probes by *in vitro* transcription according to the manufacturer’s instructions. The *in situ* hybridization experiment was performed as described in the Cold Spring Harbor Arabidopsis Molecular Genetics Course (www.Arabidopsis.org/cshl-course/5-in_situ.html). The hybridization signals were observed and photographed under Olympus BX53 microscope using SPOT color camera. All photos were treated with Adobe Photoshop CS5 software.

### *In vitro* pollen germination and pollen KI-I_2_ staining

Pollen grains from dehisced anthers (WT and RNAi plants) were placed on grass slides at 35 °C for 2 h in a pollen germination medium consisting of 1 mM CaCl_2_, 1 mM KCl, 0.8 mM MgSO_4_, 1.6 mM H_3_BO_3_, 30 mM CaSO_4_, 0.03% casein, 0.3% 2-(N-morpholino) ethanesulfonic acid, 10% sucrose and 12.5% polyethylene glycol. The humidity was maintained at above 90%. The 1% iodium potassium-iodide solution was used for pollen fertility staining. The germination pollen grains and KI-I_2_ stained pollens were observed with a microscope (Olympus, BX53) in bright-field illumination. Five fields per slide were photographed for statistics. In order to get accurate data, 20 plants from every RNAi line were selected for the experiments, and three anthers from panicle top, middle part and basal were used for analysis. The EXCEL 2010 was performed for the statistical and significance analysis.

### Plant materials and growth condition

All plants were grown under long-day conditions under natural light in Wuhan, China. ZH11 was planted as WT in this study. The dsRNA was carried out according to the method [21]. A portion of coding sequence fragment was amplified using primer set from *OsSTA28*, *OsSTA99* and *OsSTA208* cDNA clone and cloned into pMD18-T vector (Takara). The amplified fragment in the T-vector was transferred to final pDS1301 vector with KpnI/BamHI and SacI/SpeI. The final *OsSTA*-RNAi construct was transformed to Agrobacterium strain EH105A. Rice transformation was performed in the ZH11 callus, screened by hygromycin and transgenic plants were regenerated. The mature anthers before flowering were chose for gene suppressed expression verification.

### Cytology observation of anther development

The anther from sporogenous cell stage, pollen mother cell stage, meiosis stage, microspore stage, vacuolated pollen stage, bi-cellular pollen stage, and mature pollen stage were chosen for the cytological observations to identify defects in anther development. Ten plants of each RNAi line and 5 anthers per plant were paraffin sectioned for analysis. Paraffin sections were made according to the method described in a previous article [46]. The phenotypes were observed and photographed under Olympus BX53 microscope using SPOT color camera.

## Additional files

Additional file 1: Table S1. The Affymetrix microarray data of *OsSTA* genes in various tissues during rice life cycle and rice male reproductive process. Additional file 2: Table S2. Detailed information of samples used in the microarray analysis. Additional file 3: Table S3. Detailed information of genomic sequences and protein characteristics about 291 *OsSTA* genes. Additional file 4: Table S4. 
*sSTA* real-time PCR confirmed primers. Additional file 5: Figure S1. 
*In situ* localization of *OsSTA* transcripts in vegetative organs in ZH11. A7-A9: *OsSTA28*; B7-B9: *OsSTA208*; C7-C9: *OsSTA196*; D7-D9: *OsSTA99*; E7-E9: *OsSTA68*; A7-E7: root; A8-E8: stem; A9-E9: leaf. Bars = 50 μm. **Figure S2.** Significant GO annotations for genes indicated in the co-expression networks with *OsSTAs*. The boxes in the graph list the GO identifier, the statistical significance, and the description of the GO term. The color of the box indicates the significance of the term (p < 0.05). A: biological process; B: molecular function; C: cellular component. **Figure S3.** Suppressed *OsSTA* gene expression in mature anther of T_0_ RNAi plants. Y-axis represents relative expression values obtained using real-time PCR. X-axis represents the independent transgenic plants. **Figure S4.** Suppressed *OsSTA* genes expression in mature panicles and pollen fertility of T_2_ RNAi plants. X-axis represents the independent transgenic plants. Additional file 6: Table S5. GO annotations for genes indicated in the agriGO database. FDR indicates false discovery rate. P: biological process; C: cellular component; F: molecular function. Additional file 7: Table S6. The distribution of Pearson’s correlation coefficients (PCC) from 291 *OsSTA* genes and detailed information of the 1510 co-expressed genes. Additional file 8: Table S7. Detailed information of *cis*-elements in the *OsSTA* promoters and *cis*-elements for phytohormone responses.

## Abbreviations

GO: Gene ontology
RNAi: RNA interference
WT: Wild type
MH63: Minghui63
ZS97: Zhenshan97
ZH11: Zhonghua11
PCC: Pearson’s correlation coefficients
PME: Pectin methylesterase
PMEI: Pectin methylesterase inhibitor
