# Supplementary material for: The mature anther-preferentially expressed genes are associated with pollen fertility, pollen germination and anther dehiscence in rice
Source: BMC Genomics. 2015 Feb 19;16(1):101. doi: 10.1186/s12864-015-1305-y (PMC4340671; doi:10.1186/s12864-015-1305-y)
Supplement: Additional file 5: Figure S1. — In situ localization of OsSTA transcripts in vegetative organs in ZH11. A7-A9: OsSTA28; B7-B9: OsSTA208; C7-C9: OsSTA196; D7-D9: OsSTA99; E7-E9: OsSTA68; A7-E7: root; A8-E8: stem; A9-E9: leaf. Bars = 50 μm. Figure S2. Significant GO annotations for genes indicated in the co-expression networks with OsSTAs. The boxes in the graph list the GO identifier, the statistical significance, and the description of the GO term. The color of the box indicates the significance of the term (p < 0.05). A: biological process; B: molecular function; C: cellular component. Figure S3. Suppressed OsSTA gene expression in mature anther of T0 RNAi plants. Y-axis represents relative expression values obtained using real-time PCR. X-axis represents the independent transgenic plants. Figure S4. Suppressed OsSTA genes expression in mature panicles and pollen fertility of T2 RNAi plants. X-axis represents the independent transgenic plants. [file 12864_2015_1305_MOESM5_ESM.pdf]

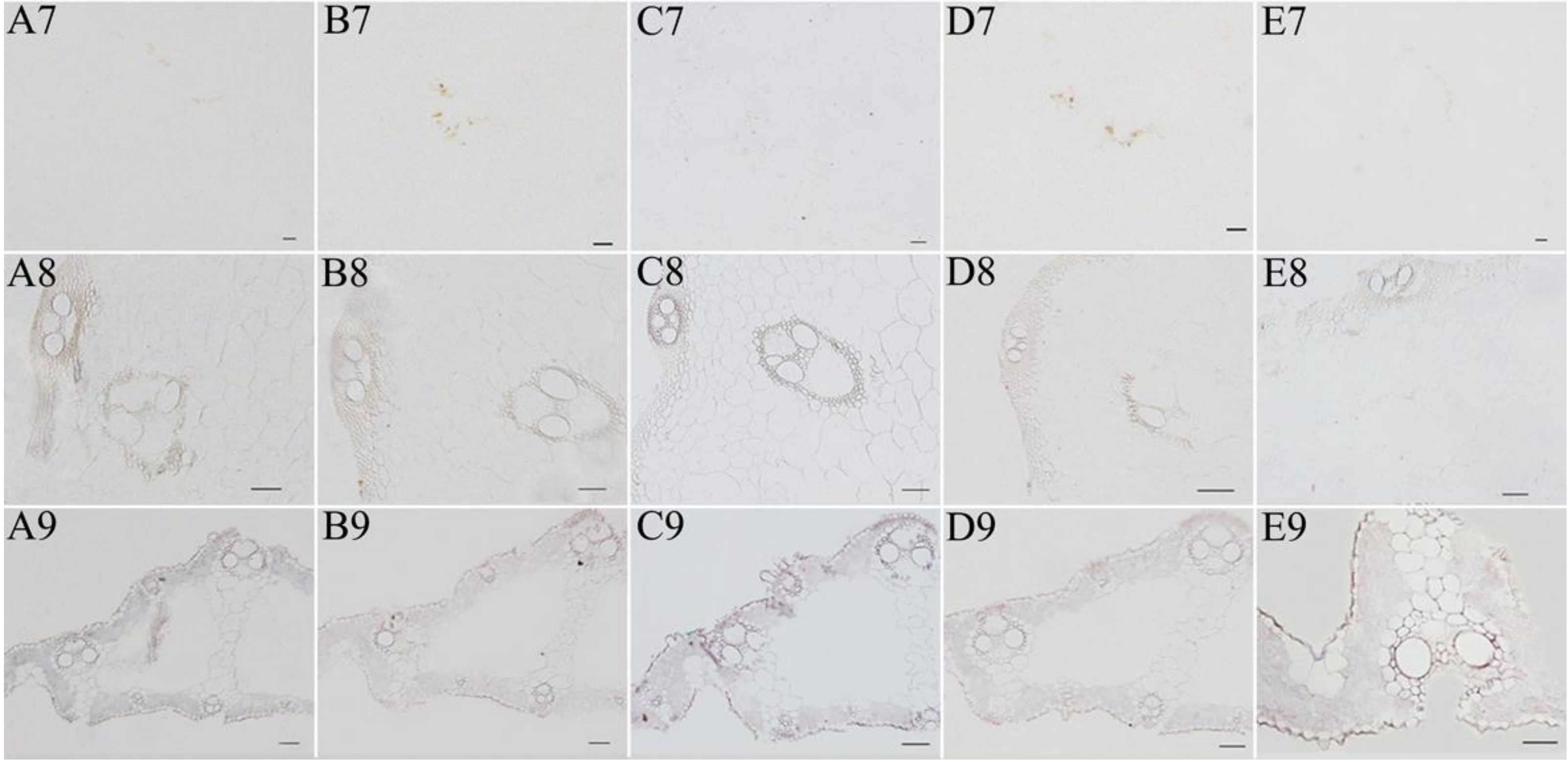

Figure S1 In situ localization of *OsSTA* genes transcript in vegetative organs in ZH11. A7-A9: *OsSTA28*; B7-B9: *OsSTA208*; C7-C9: *OsSTA196*; D7-D9: *OsSTA99*; E7-E9: *OsSTA68*; A7-E7: root; A8-E8: stem; A9-E9: leaf. Bars = 50um.

A

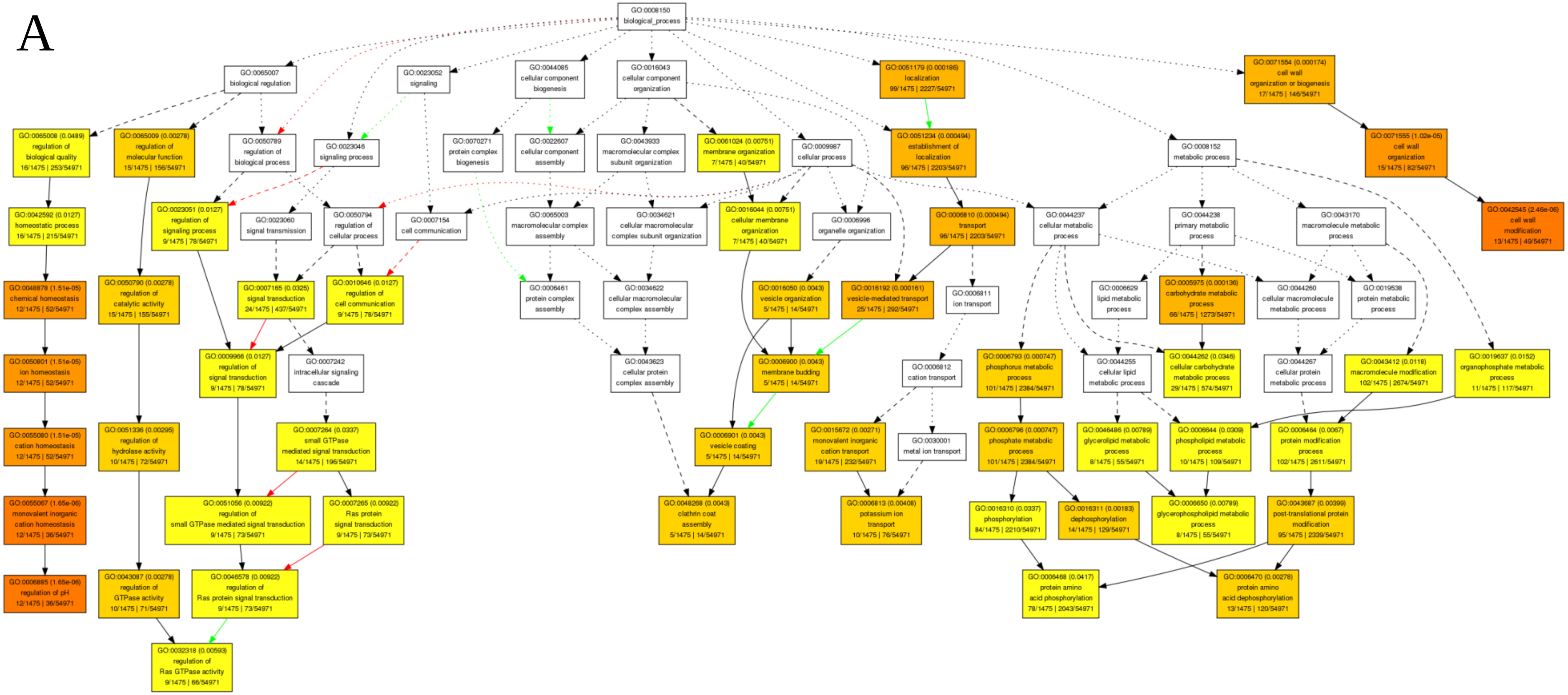
GO:0008150 biological_process
GO:0065007 biological regulation
GO:0023052 signaling
GO:0044085 cellular component biogenesis
GO:0016043 cellular component organization
GO:0051179 (0.000186) localization 99/1475 | 2227/54971
GO:0071554 (0.000174) cell wall organization or biogenesis 17/1475 | 146/54971
GO:0065008 (0.0489) regulation of biological quality 16/1475 | 253/54971
GO:0065009 (0.00278) regulation of molecular function 15/1475 | 156/54971
GO:0050789 regulation of biological process
GO:0023046 signaling process
GO:0070271 protein complex biogenesis
GO:0022607 cellular component assembly
GO:0043933 macromolecular complex subunit organization
GO:0061024 (0.00751) membrane organization 7/1475 | 40/54971
GO:0009987 cellular process
GO:0051234 (0.000494) establishment of localization 96/1475 | 2203/54971
GO:0008152 metabolic process
GO:0071555 (1.02e-05) cell wall organization 15/1475 | 82/54971
GO:0042592 (0.0127) homeostatic process 16/1475 | 215/54971
GO:0023051 (0.0127) regulation of signaling process 9/1475 | 78/54971
GO:0023060 signal transmission
GO:0050794 regulation of cellular process
GO:0007154 cell communication
GO:0065003 macromolecular complex assembly
GO:0034621 cellular macromolecular complex subunit organization
GO:0016044 (0.00751) cellular membrane organization 7/1475 | 40/54971
GO:0006996 organelle organization
GO:0006810 (0.000494) transport 96/1475 | 2203/54971
GO:0044237 cellular metabolic process
GO:0044238 primary metabolic process
GO:0043170 macromolecule metabolic process
GO:0042545 (2.46e-06) cell wall modification 13/1475 | 49/54971
GO:0048878 (1.51e-05) chemical homeostasis 12/1475 | 52/54971
GO:0050790 (0.00278) regulation of catalytic activity 15/1475 | 155/54971
GO:0007165 (0.0325) signal transduction 24/1475 | 437/54971
GO:0010646 (0.0127) regulation of cell communication 9/1475 | 78/54971
GO:0006461 protein complex assembly
GO:0034622 cellular macromolecular complex assembly
GO:0016050 (0.0043) vesicle organization 5/1475 | 14/54971
GO:0016192 (0.000161) vesicle-mediated transport 25/1475 | 292/54971
GO:0006811 ion transport
GO:0006629 lipid metabolic process
GO:0005975 (0.000136) carbohydrate metabolic process 66/1475 | 1273/54971
GO:0044260 cellular macromolecule metabolic process
GO:0019538 protein metabolic process
GO:0050801 (1.51e-05) ion homeostasis 12/1475 | 52/54971
GO:0009966 (0.0127) regulation of signal transduction 9/1475 | 78/54971
GO:0007242 intracellular signaling cascade
GO:0043623 cellular protein complex assembly
GO:0006900 (0.0043) membrane budding 5/1475 | 14/54971
GO:0006812 cation transport
GO:0006793 (0.000747) phosphorus metabolic process 101/1475 | 2384/54971
GO:0044255 cellular lipid metabolic process
GO:0044262 (0.0346) cellular carbohydrate metabolic process 29/1475 | 574/54971
GO:0044267 cellular protein metabolic process
GO:0043412 (0.0118) macromolecule modification 102/1475 | 2674/54971
GO:0019637 (0.0152) organophosphate metabolic process 11/1475 | 117/54971
GO:0055080 (1.51e-05) cation homeostasis 12/1475 | 52/54971
GO:0051336 (0.00295) regulation of hydrolase activity 10/1475 | 72/54971
GO:0007264 (0.0337) small GTPase mediated signal transduction 14/1475 | 196/54971
GO:0006901 (0.0043) vesicle coating 5/1475 | 14/54971
GO:0015672 (0.00271) monovalent inorganic cation transport 19/1475 | 232/54971
GO:0030001 metal ion transport
GO:0006796 (0.000747) phosphate metabolic process 101/1475 | 2384/54971
GO:0046486 (0.00789) glycerolipid metabolic process 8/1475 | 55/54971
GO:0006644 (0.0309) phospholipid metabolic process 10/1475 | 109/54971
GO:0006464 (0.0067) protein modification process 102/1475 | 2611/54971
GO:0055067 (1.65e-06) monovalent inorganic cation homeostasis 12/1475 | 36/54971
GO:0051056 (0.00922) regulation of small GTPase mediated signal transduction 9/1475 | 73/54971
GO:0007265 (0.00922) Ras protein signal transduction 9/1475 | 73/54971
GO:0048268 (0.0043) clathrin coat assembly 5/1475 | 14/54971
GO:0006813 (0.00408) potassium ion transport 10/1475 | 76/54971
GO:0016310 (0.0337) phosphorylation 84/1475 | 2210/54971
GO:0016311 (0.00183) dephosphorylation 14/1475 | 129/54971
GO:0006650 (0.00789) glycerophospholipid metabolic process 8/1475 | 55/54971
GO:0043687 (0.00399) post-translational protein modification 95/1475 | 2339/54971
GO:0006885 (1.65e-06) regulation of pH 12/1475 | 36/54971
GO:0043087 (0.00278) regulation of GTPase activity 10/1475 | 71/54971
GO:0046578 (0.00922) regulation of Ras protein signal transduction 9/1475 | 73/54971
GO:0006468 (0.0417) protein amino acid phosphorylation 78/1475 | 2043/54971
GO:0006470 (0.00278) protein amino acid dephosphorylation 13/1475 | 120/54971
GO:0032318 (0.00593) regulation of Ras GTPase activity 9/1475 | 66/54971

B

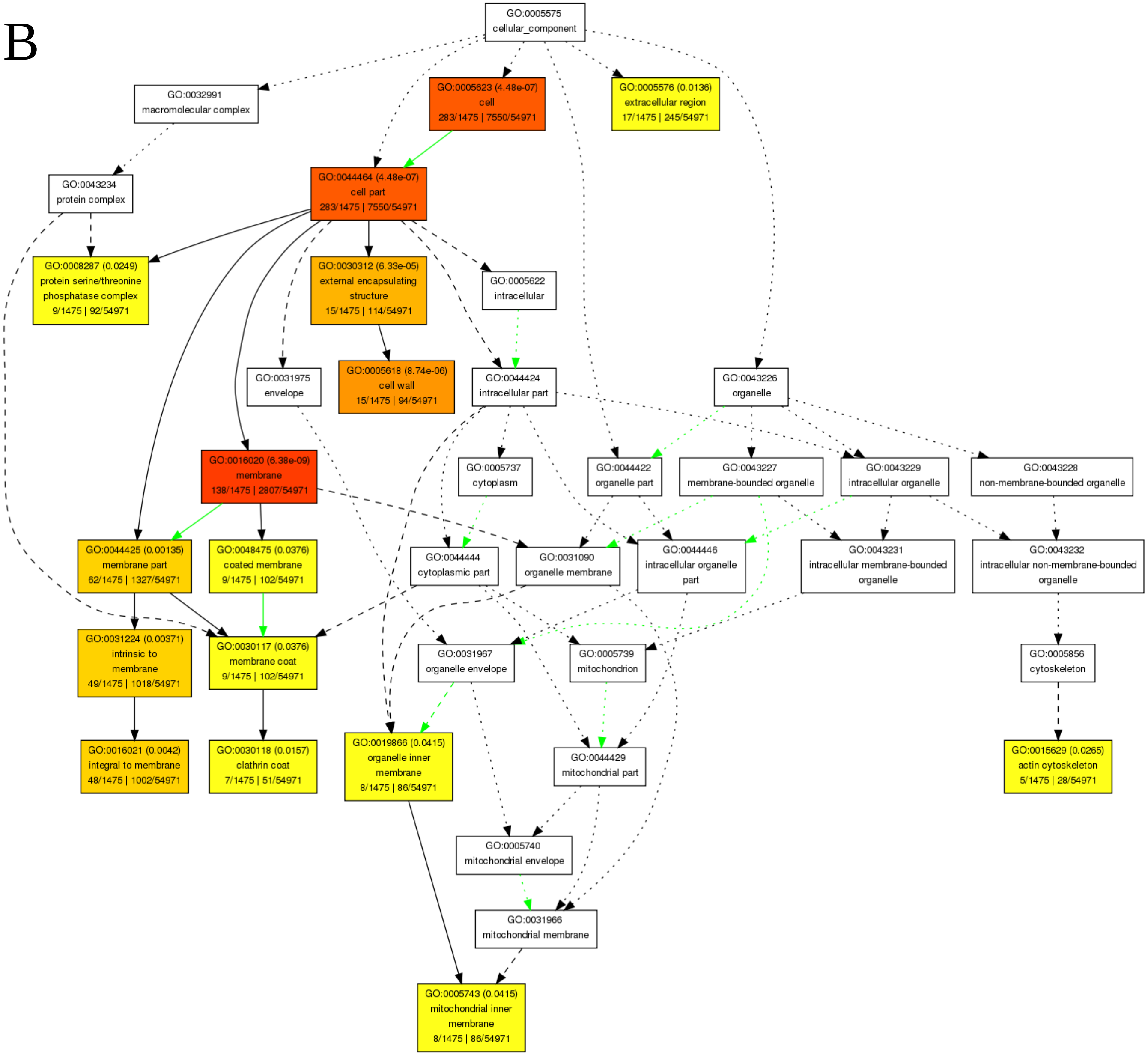
GO:0005575
cellular_component
GO:0032991
macromolecular complex
GO:0005623 (4.48e-07)
cell
283/1475 | 7550/54971
GO:0005576 (0.0136)
extracellular region
17/1475 | 245/54971
GO:0043234
protein complex
GO:0044464 (4.48e-07)
cell part
283/1475 | 7550/54971
GO:0008287 (0.0249)
protein serine/threonine
phosphatase complex
9/1475 | 92/54971
GO:0030312 (6.33e-05)
external encapsulating
structure
15/1475 | 114/54971
GO:0005622
intracellular
GO:0031975
envelope
GO:0005618 (8.74e-06)
cell wall
15/1475 | 94/54971
GO:0044424
intracellular part
GO:0043226
organelle
GO:0016020 (6.38e-09)
membrane
138/1475 | 2807/54971
GO:0005737
cytoplasm
GO:0044422
organelle part
GO:0043227
membrane-bounded organelle
GO:0043229
intracellular organelle
GO:0043228
non-membrane-bounded organelle
GO:0044425 (0.00135)
membrane part
62/1475 | 1327/54971
GO:0048475 (0.0376)
coated membrane
9/1475 | 102/54971
GO:0044444
cytoplasmic part
GO:0031090
organelle membrane
GO:0044446
intracellular organelle
part
GO:0043231
intracellular membrane-bounded
organelle
GO:0043232
intracellular non-membrane-bounded
organelle
GO:0031224 (0.00371)
intrinsic to
membrane
49/1475 | 1018/54971
GO:0030117 (0.0376)
membrane coat
9/1475 | 102/54971
GO:0031967
organelle envelope
GO:0005739
mitochondrion
GO:0005856
cytoskeleton
GO:0016021 (0.0042)
integral to membrane
48/1475 | 1002/54971
GO:0030118 (0.0157)
clathrin coat
7/1475 | 51/54971
GO:0019866 (0.0415)
organelle inner
membrane
8/1475 | 86/54971
GO:0044429
mitochondrial part
GO:0015629 (0.0265)
actin cytoskeleton
5/1475 | 28/54971
GO:0005740
mitochondrial envelope
GO:0031966
mitochondrial membrane
GO:0005743 (0.0415)
mitochondrial inner
membrane
8/1475 | 86/54971

C

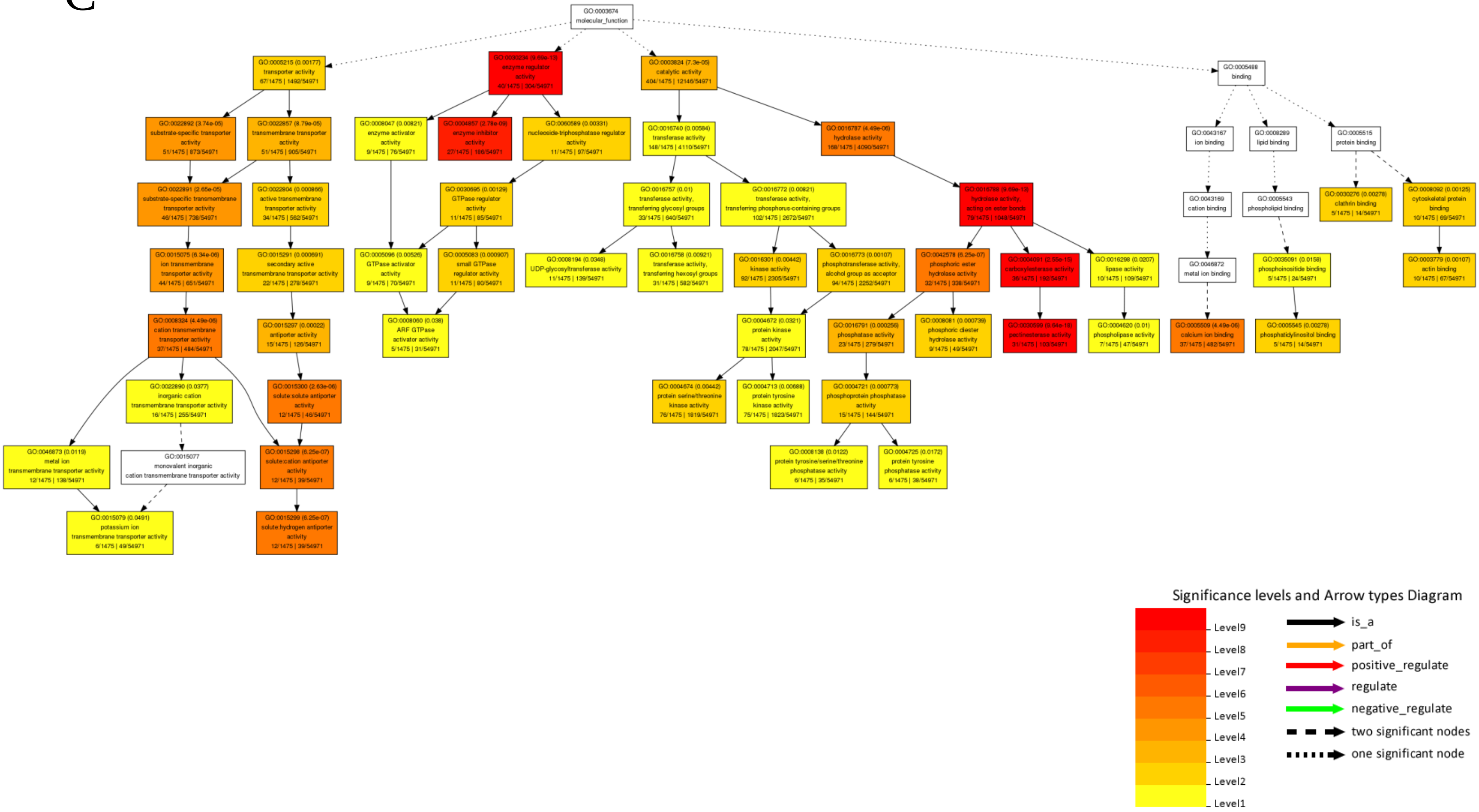

Figure S2 Significant GO annotations for genes indicated in the co-expression networks with *OsSTAs*. The boxes in the graph list the GO identifier, the statistical significance, and the description of the GO term. The color of the box indicates the significance of the term ($p < 0.05$). A: biological process; B: cellular component; C: molecular function.

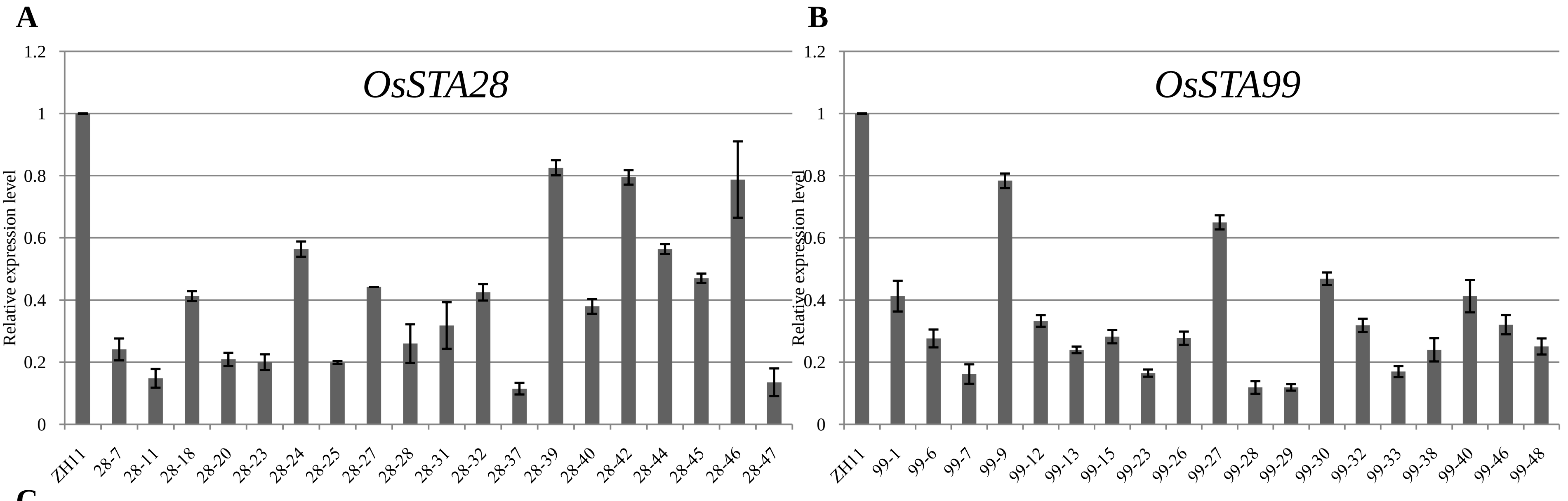

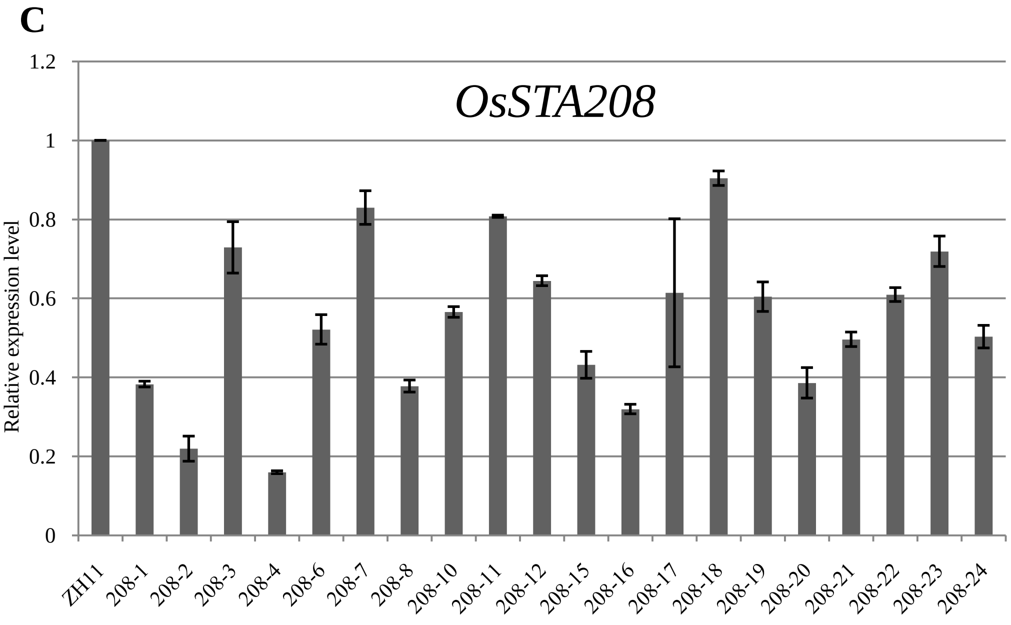

Figure S3 Suppressed *OsSTA* genes expression in mature panicles of T_0 RNAi plants. Y-axis represents relative expression values obtained using QPCR. X-axis represent the independent transgenic plants.

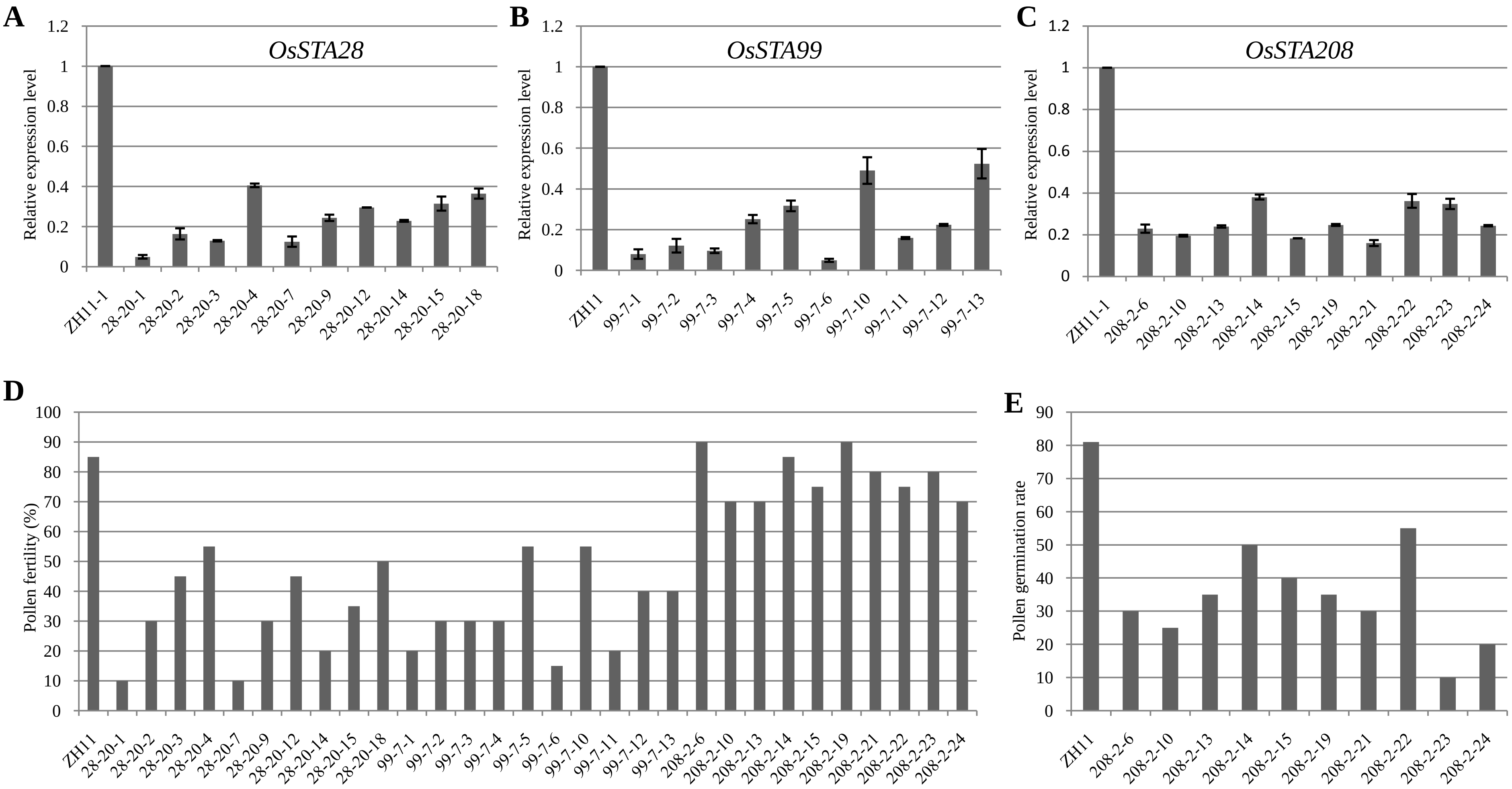

Figure S4 Suppressed *OsSTA* genes expression in mature panicles and pollen fertility of T_2 RNAi plants. X-axis represents the independent transgenic plants.
